# Lymphangioleiomyomatosis: A Case Report and Review of Literature

**DOI:** 10.7759/cureus.3938

**Published:** 2019-01-22

**Authors:** Jee Ah Rhee, Ajay Adial, Rammohan Gumpeni, Asma Iftikhar

**Affiliations:** 1 Internal Medicine, New York - Presbyterian Hospital Queens, Flushing, USA

**Keywords:** cystic lung disease, pneumothorax, lymphangioleiomyomatosis

## Abstract

Pulmonary lymphangioleiomyomatosis (LAM) is a disease, which is most commonly seen in women of childbearing age. The objective of this article was to provide education about the typical clinical presentation, radiologic findings, histology, treatment approaches, and differential diagnosis. Pulmonary LAM is a cystic lung disease, usually generalized and progressive and extremely difficult to treat and is considered to have a poor prognosis. Patients with LAM often present with an insidious onset of dyspnea; this could be secondary to pneumothorax. However, it could also be present as chylothorax and hemoptysis. We discussed a case who presented with chest pain and shortness of breath due to pneumothorax and retrospectively diagnosed with LAM.

## Introduction

Lymphangioleiomyomatosis (LAM) is a multisystem disorder characterized by the proliferation of smooth muscle cells that result in cystic lung disease as well as extrapulmonary manifestations such as angiomyolipomas and lymphatic tumors. It predominantly occurs in pre-menopausal women but could be present in postmenopausal women, presenting most commonly with progressive dyspnea and spontaneous pneumothorax. Once thought to be a fatal disease for young women, with the only treatment option being lung transplant, the definition of classical LAM is actively evolving, thanks to a growing accumulation of data on LAM with international registries, which may help catalyze the advancement in diagnostic studies and therapeutic options.

## Case presentation

**Figure 1:**
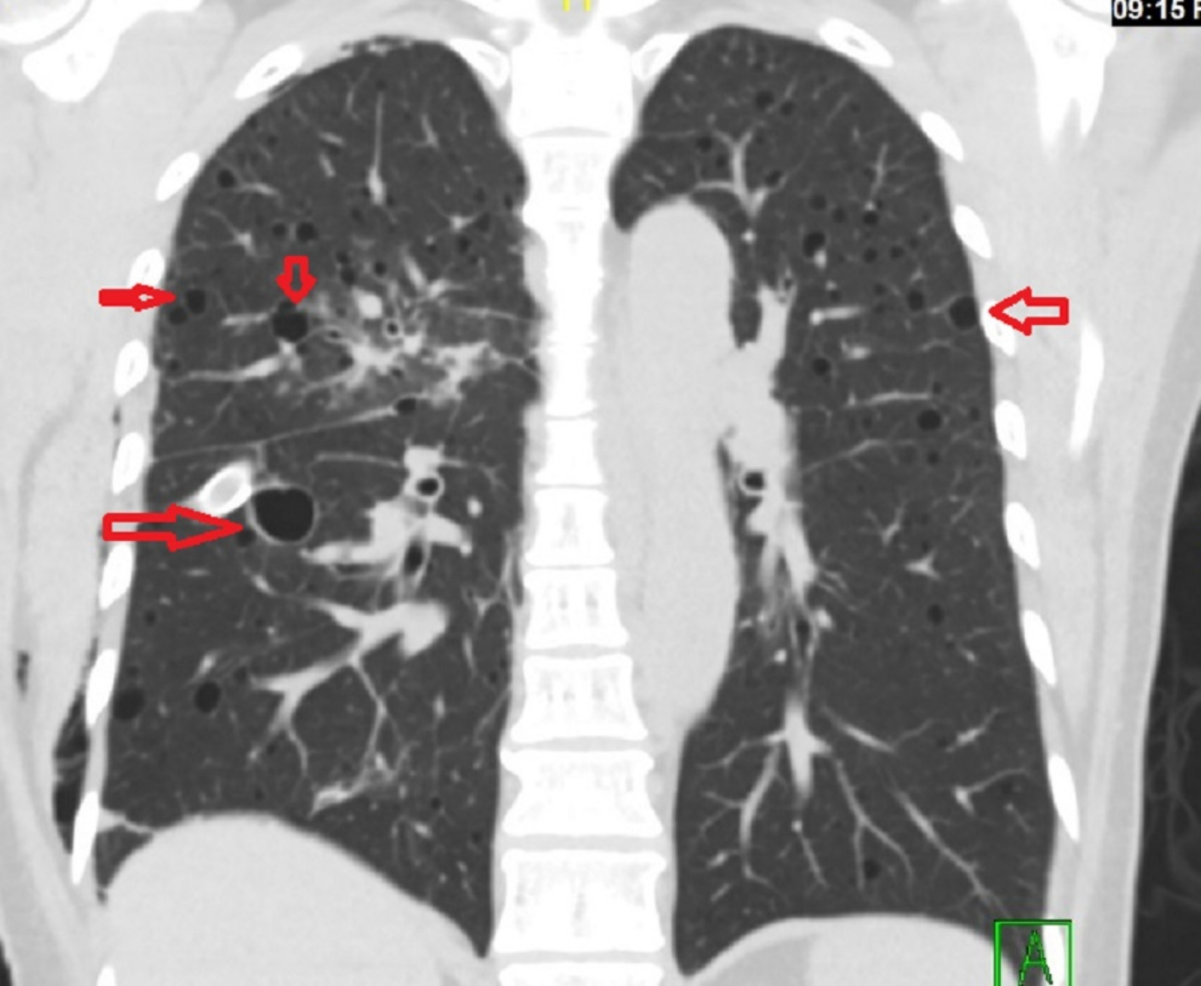
Coronal section: Chest computed tomography scan showing multiple cysts

**Figure 2:**
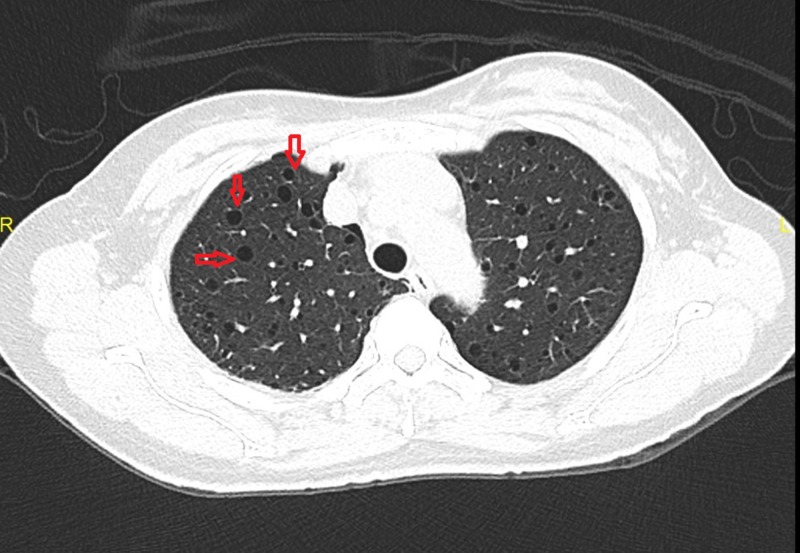
Sagittal section: Chest computed tomography scan showing multiple cysts

A 55-year-old woman presented three days after a sudden onset of right-sided chest pain, pleuritic and positional in nature, associated with an acute onset of shortness of breath. She had gone to her primary care physician, who performed a chest X-ray and urged her to come to the hospital. Upon presentation at the emergency department, her oxygen saturation was above 95% on room air, and she was not in any respiratory distress, but her exam was significant for decreased breath sound on the right. A chest X-ray confirmed a large right-sided pneumothorax with small pleural effusion. A chest tube was inserted on the right side for the resolution of the pneumothorax, and subsequent computed tomography (CT) scan of the chest revealed bilateral diffuse bullous disease of the lung with multiple cysts (Figure 1-2). The patient underwent video-assisted thoracoscopic surgery for right thoracoscopic wedge resection of a lung bleb and talc pleurodesis. Gross examination of the specimen revealed several dilated air-like spaces ranging from 0.2 cm to 0.4 cm in size. The hospital course was complicated by postsurgical pneumonia, but she recovered fully and was discharged to home with only minimal symptoms of dyspnea on exertion. Upon further investigations, she was found to have multiple small lesions of angiomyolipoma on the right kidney with diffuse retroperitoneal lymphadenopathy. One of the lymph nodes was biopsied, and pathology revealed predominantly spindle cells positive for HHF35 and smooth muscle actin, consistent with the diagnosis of leiomyoma. At the eight-month follow-up at the pulmonology clinic, her pulmonary function test (PFT) showed normal vital capacity and forced expiratory volume in one second (FEV1), but moderately reduced diffusion capacity, which may also be related to LAM. At her 12-month and 24-month follow-up visits, her PFT results showed improvements in peak flow and diffusion capacity, and the patient continues to report no symptoms other than minimal dyspnea on exertion.

## Discussion

Our patient represents a classic presentation of LAM in a woman of menopausal age; LAM is historically considered a rare and fatal disease of women of childbearing age. Pathology of this rare disease is characterized by abnormal proliferation of smooth muscle cells, most notably in the lung parenchyma and airway walls as well as the lymphatics [1-2]. As the disease progresses, it leads to narrowing and obstruction of the airway that presents similarly to obstructive lung disease, but ultimately resulting in alveolar damage and the development of cystic disease of the lungs as well as the lymphatic system. Patients with severe LAM in whom diffusing capacity of the lungs for carbon monoxide (DLCO) and/or FEV1 have decreased to less than 40% of predicted who require continuous oxygen therapy should refer for lung transplantation [3].

The estimated prevalence of LAM is thought to be around one to 2.6 patients in 1,000,000 in the general female population [4]. However, due to the insidious nature of the disease progression in LAM and the lack of specific laboratory tests for the diagnosis, the incidence of disease is probably underestimated.

The clinical presentation of LAM greatly varies but most commonly includes dyspnea on exertion. Other common physical findings of LAM include spontaneous pneumothorax (57%), hemoptysis (32%), abdominal angiolipomas (32%), lymphangioleiomyoma (29%), and pleural effusions (12%), and less commonly, chylothorax, chylous ascites, chyluria, chyloptysis, and abdominal hemorrhage caused by renal angiolipomas [5]. These symptoms typically present in the later stages of the disease, and therefore, the initial presentation of LAM is often mistaken for symptoms of reactive airway disease. Therefore, patients are often misdiagnosed and usually treated with bronchodilators [1]. Patients with LAM are at a higher risk of spontaneous pneumothorax due to the proliferation of smooth muscles in the bronchioles and subsequent narrowing of the airway and air trapping that leads to diffuse cystic lung disease [3]. One study reported 40% to 80% of LAM patients can have recurrent pneumothoraces [2].

The involvement of the smooth muscle cells of the pulmonary venous vasculature may lead to hemosiderin deposition in the lung parenchyma due to recurrent hemorrhage, but clinically significant hemoptysis in LAM is present only in about 32% of patients [2,5]. Renal angiolipomas could be incidental findings in up to 50% of patients [6]. Radiographic findings often vary depending on disease severity and progression. Typically, chest X-rays are notable for hyperinflated lungs due to the obstructive nature of LAM, and CT shows the diffuse cystic lung parenchyma. It may also show a ground-glass appearance from hemosiderosis [7]. A reticular pattern on X-ray may be present in later stages of LAM from the coalescence of the cysts, but this must be differentiated from Langerhans’ cell histiocytosis, which may also present with similar symptoms of the pulmonary and lymphatic disease and the reticular pattern on X-rays [8].

Concomitant with the chest X-ray findings of hyperinflated lungs, PFT in patients with LAM is often obstructive or mixed pattern [1,7]. As in obstructive lung diseases, total lung capacity (TLC) is often increased due to air-trapping, and residual volume (RV), as well as the RV/TLC ratio, is also increased. Airflow is limited with reduced FEV1, and this may be due to not only the increased airway resistance but also the decreased lung elasticity [8]. About 20% of patients with LAM demonstrate improvement with bronchodilators. LAM patients may have near-normal PFTs at rest, and only with exercise testing, they would reveal abnormalities in ventilation and gas exchange with hypoxemia. However, disease progression can be best monitored with diffusion capacity and FEV1 [9].

LAM should be in the differential diagnosis of any female patients with these symptoms or findings. The diagnosis can be made with high-resolution CT scans, but in most cases, a tissue biopsy is obtained by various means, and the diagnosis is confirmed with the characteristic immunochemical stains that are specific for smooth muscle cells (e.g., actin, desmin, or HMB-45). Among these stains, HMB-45 is the gold standard that is specific for the atypical smooth muscle cells of LAM [10].

The fact that LAM presents predominantly in premenopausal women and never before menarche has led to numerous studies to determine the role of estrogens in the pathogenesis of LAM [11]. Estrogen and progesterone receptors were found in the smooth muscle cells of the lungs and angiomyolipomas, and worsening pulmonary function was noted during pregnancy or estrogen therapy [12-13]. Moreover, the disease progression is shown to subside after oophorectomy or menopause. These studies highlight the significant association in the disease progression with progesterone, and no association has been established, and hence, there is yet no definite therapeutic strategies targeting the hormonal receptors [14].

**Figure 3:**
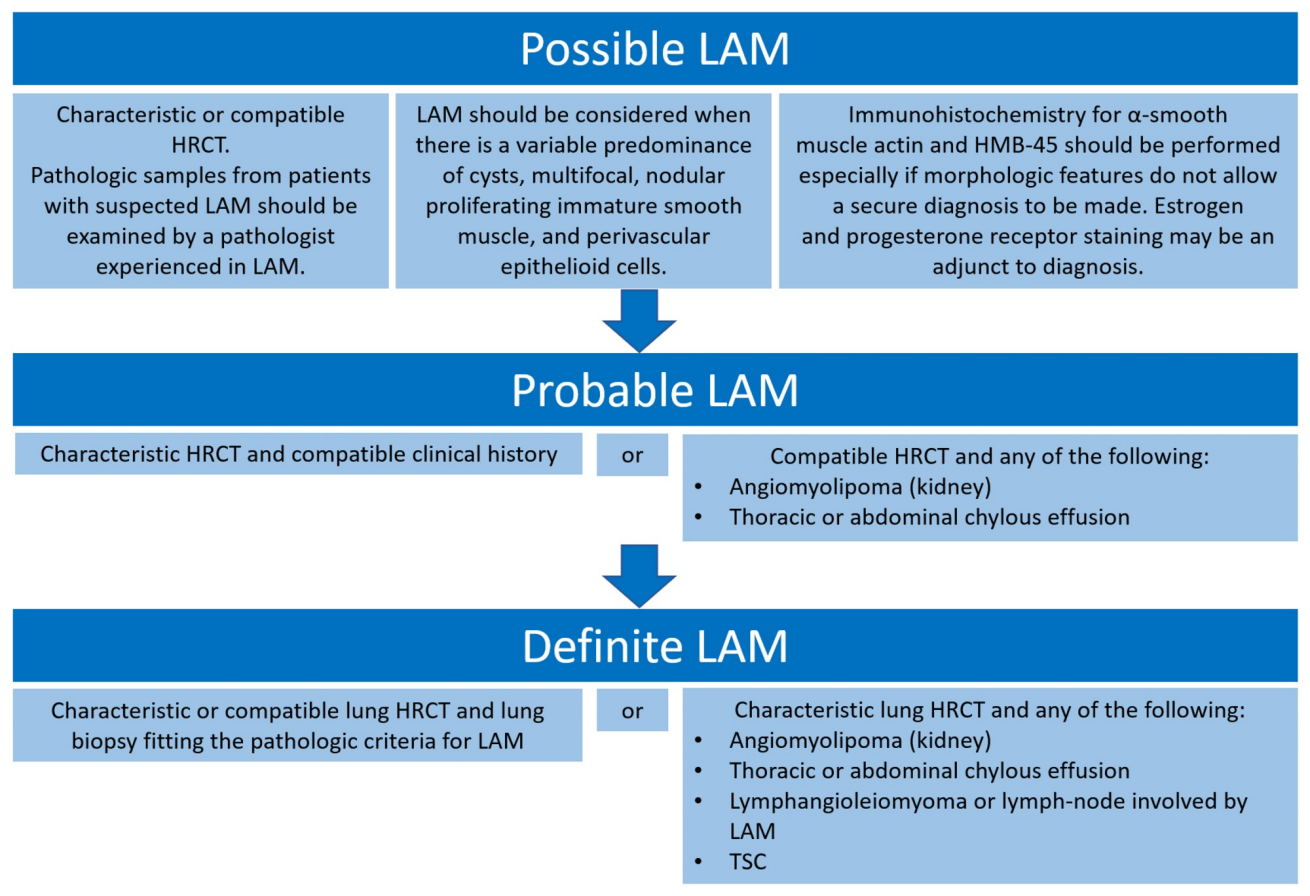
Diagnostic criteria for LAM LAM: lymphangioleiomyomatosis

Bronchodilators are part of the supportive measures in LAM patients with dyspnea and sometimes are the only treatment LAM patients require. Depending on the disease severity, some patients are started on sirolimus or everolimus, immune-modulating therapies that target the mammalian rapamycin (mTOR) signaling pathway by inhibiting the mTOR complex, which provides a median transplant-free survival of approximately 29 years from the onset of symptoms and 10-year transplant-free survival of 86% [15-16]. It should be noted that these therapeutic options are only stabilizing and not curative, and lung transplantation remains the last treatment option for patients with advanced LAM for improvement in their quality of life. Diagnostic criteria have been ESR as outlined below (Figure 3) [17].

## Conclusions

LAM is a disease of women traditionally of reproductive age, but it could be present in patients in the menopause age group. LAM should be in the differential diagnosis of cystic disease and spontaneous pneumothorax. Various chemotherapy options are available, which can slow the progress of LAM. Nevertheless, lung transplantation remains the definitive treatment.
